# Efficacy and safety of front-line treatment regimens for Waldenstrom macroglobulinaemia: a systematic review and meta-analysis

**DOI:** 10.1038/s41408-023-00916-5

**Published:** 2023-09-07

**Authors:** Wee-Lee Chan, Vanessa Cui Lian Chong, Ian Jun Yan Wee, Li Mei Poon, Esther Hian Lee Chan, Joanne Lee, Yen-Lin Chee, Anand D. Jeyasekharan, Wee-Joo Chng, Miny Samuel, Sanjay de Mel

**Affiliations:** 1 Department of Haematology-Oncology, National University Cancer Institute, Singapore, Singapore; 2 Department of Haematology, Tan Tock Seng Hospital, Singapore, Singapore; 3 Department of Surgery, Singapore General Hospital, Singapore, Singapore; 4 Yong Loo Lin School of Medicine, National University of Singapore, Singapore, Singapore; 5 Cancer Science Institute of Singapore, National University of Singapore, Singapore, Singapore; 6 Research Support Unit, National University of Singapore, Singapore, Singapore

**Keywords:** Translational research, B-cell lymphoma

## Abstract

Rituximab-based chemo-immunotherapy is currently the standard first-line treatment for Waldenstrom macroglobulinaemia (WM), while ibrutinib has emerged as an alternative. In the absence of randomised trials (RCTs) comparing these regimens, the optimal first-line treatment for WM remains uncertain. In this systematic review and meta-analysis, we sought to assess the efficacy and safety of first-line treatment regimens for WM. We searched key databases from January 2007 to March 2023, including phase II and III trials, including treatment-naïve WM patients treated with rituximab-based regimens or ibrutinib. Response rates, progression-free survival (PFS), overall survival (OS), and toxicities were evaluated. Four phase III and seven phase II trials were included among 736 unique records. Pooled response rates from all comparative and non-comparative trials were 46%, 33% and 26% for bendamustine rituximab (BR), bortezomib-dexamethasone, cyclophosphamide, rituximab (BDRC) and ibrutinib rituximab (IR), respectively. Two-year pooled PFS was 89%, 81% and 82% with BR, BDRC and IR, respectively. Neuropathy was more frequent with bortezomib, while haematologic and cardiac toxicities were more common with chemo-immunotherapy and ibrutinib-based regimens respectively. Our findings suggest that BR yields higher response rates than bortezomib or ibrutinib-based combinations. RCTs comparing BR against emerging therapies, including novel Bruton Tyrosine Kinase Inhibitors, are warranted.

## Introduction

Waldenstrom macroglobulinaemia (WM) is a rare mature B-cell neoplasm characterised by clonal lymphoplasmacytic (LPL) bone marrow (BM) infiltration and immunoglobulin M (IgM) paraproteinaemia [1]. While a watch-and-wait approach is appropriate for asymptomatic patients, treatment is indicated when clinical manifestations arise due to the IgM paraprotein or LPL infiltrate [2]. Major classes of agents commonly used for the treatment of WM include monoclonal antibodies, alkylating agents, proteasome inhibitors, and Bruton Tyrosine Kinase inhibitors (BTKi) [3].

Rituximab is a key component of WM treatment, and has been evaluated in combination with bortezomib [4, 5], dexamethasone and bortezomib (BDR) [6, 7]; dexamethasone and cyclophosphamide (DRC) [8], bortezomib cyclophopshamide dexamethasone(B-DRC) [9], and bendamustine (BR) [10–12]. The BTKi ibrutinib was shown to be an effective therapy for newly diagnosed WM and is approved by the Food and Drug Administration for this indication [13]. While oral administration and a favourable toxicity profile have made ibrutinib an attractive option, fixed-duration treatment with chemo-immunotherapy remains a valid alternative [2, 14].

The rarity of WM and the paucity of randomised trials (RCTs) comparing chemo-immunotherapy and BTKi-based treatment has resulted in uncertainty regarding the optimal therapy for treatment-naïve WM patients. In this systematic review and meta-analysis, our objective was to compare the efficacy and safety of rituximab-based chemo-immunotherapy and BTKi-based regimens for newly diagnosed WM from all comparative and non-comparative trials.

## Methods

A systematic literature review was performed according to the “Preferred Reporting Items for Systematic Reviews and Meta-Analyses (PRISMA) guidelines [15]. A study protocol was written a priori and registered with PROSPERO (Registration number: 283550).

We performed literature searches of the following electronic databases: Medline and Medline In-process (using the PubMed platform), Embase (using the Elsevier Platform), and The Cochrane Library from January 2007 to March 2023. The rationale for the period of the search strategy was that the key trials of rituximab-based chemo-immunotherapy and BTKi therapy were published after 2006. We also searched ClinicalTrials.gov and the International Clinical Trials Registry Platform (http://www.who.int/ctrp/en/.) to identify ongoing, discontinued and completed clinical trials: There was no limit on language or geographical perspective. Briefly, the population studied was treatment-naïve WM patients, and the interventions included rituximab-based chemo-immunotherapy or BTKi-based regimens. Detailed inclusion and exclusion criteria are presented in Supplementary Tables S1 and S2.

The study selection process was performed in the following two phases: Level 1 screening: titles and abstracts of studies identified from the electronic databases and the internet searches were reviewed by two researchers (SDM, WLC), independently and in parallel, to determine eligibility according to the criteria in Supplementary Table S1. Level 2 screening: Full texts of studies selected at level 1 were obtained and reviewed by two researchers (SDM, WLC), independently and in parallel, to determine eligibility according to the criteria in Supplementary Table S2. Where there was disagreement about study relevance at either stage of screening, consensus was reached with a third member of the study team (MS).

The results of the systematic literature review were summarised qualitatively and quantitatively as appropriate. The outcomes of interest were: response rates based on International Waldenstrom Macroglobulinaemia Working Group (IWWM) criteria [16] (Supplementary Table S3), time to achievement of partial response or complete response, rates of relapse, progression-free survival (PFS), overall survival (OS), treatment-related complications, and quality-of-life scores. Where outcomes were reported as dichotomous data in trials, we calculated the risk ratios (RR) and 95% confidence interval (CI) for meta-analysis using Review Manager software (version 5.4). Evidence from single-arm studies was summarised using descriptive summary statistics. Continuous measures were expressed in the form of mean (standard deviation).

Where the demographics of the study populations and inclusion/exclusion were relatively similar between the single-arm cohort studies, a meta-analysis of proportions (expressed as a percentage), with their 95% CI, was performed using the software Comprehensive Meta-analysis (version 3.3). To establish the variance of raw proportions, a Freeman-Tukey transformation was applied. To incorporate heterogeneity (anticipated among the included studies), transformed proportions were combined using DerSimonian-Laird random effects models.

In order to assess whether effect sizes were consistent across the included studies, heterogeneity was quantified. The test for heterogeneity was performed using the I^2^ statistic, which provides a magnitude of variability, where 0% indicates that any variability is due to chance, whilst higher I^2^ values (>50%) indicate increasing levels of unexplained variability. Where we have judged that the included trials are too clinically heterogeneous to warrant a formal meta-analysis, we presented the results of the included trials in a narrative format instead of performing a meta-analysis. All the studies were evaluated with the critical appraisal tools by the Joanna Briggs Institute or the Centre for Reviews and Dissemination (Table 1).

**Table 1:** Study quality and risk of bias.

Trial number/acronym	NCT00981708	NCT00422799	NCT00250926	NCT012029730	NCT01929265 (INFL09)	FILO	NCT01788020
Is it clear in the study what is the ‘cause’ and what is the ‘effect’ (i.e., there is no confusion about which variable comes first)?	Y	Y	Y	Y	Y	Y	Y
Were the participants included in any comparisons similar?	NA	NA	NA	NA	NA	NA	NA
Were the participants included in any comparisons receiving similar treatment/care other than the exposure or intervention of interest?	NA	NA	NA	NA	NA	NA	NA
Was there a control group?	N	N	N	N	N	N	N
Were there multiple measurements of the outcome both pre- and post-intervention/exposure?	Y	Y	Y	Y	Y	Y	Y
Was follow-up complete, and if not, were differences between groups in terms of their follow-up adequately described and analysed?	Y	Y	Y	Y	Y	Y	Y
Were the outcomes of participants included in any comparisons measured in the same way?	NA	NA	NA	NA	NA	NA	NA
Were outcomes measured in a reliable way?	Y	Y	Y	Y	Y	Y	Y
Was appropriate statistical analysis used?	Y	Y	Y	Y	Y	Y	Y

Trial number/acronym	NCT00991211 (STIL)	NCT00877006 (BRIGHT)	NCT02165397 (INNOVATE)	NCT01788020
Was randomisation carried out appropriately?	Y	Y	Y	Y
Was the concealment of treatment allocation adequate?	NA	NA	NA	NA
Were the groups similar at the outset of the study in terms of prognostic factors?	Y	Y	Y	Y
Were the care providers, participants and outcome assessors blind to treatment allocation?	N	N	N	N
Were there any unexpected imbalances in drop-outs between groups?	N	N	N	N
Is there any evidence to suggest that the authors measured more outcomes than they reported?	N	N	N	N
Did the analysis include an intention-to-treat analysis? If so, was this appropriate and were appropriate methods used to account for missing data?	N	Y,Y	N	Y

Adapted from Centre for Reviews and Dissemination (2008) Systematic reviews. CRD’s guidance for undertaking reviews in health care. York: Centre for Reviews and Dissemination.

## Results

### Results of the search

**Fig. 1:**
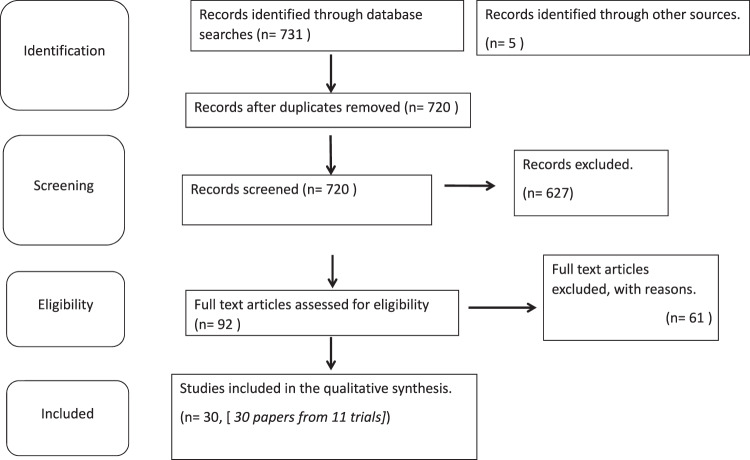
Preferred Reporting Items for Systematic Reviews and Meta-Analyses (PRISMA) chart for the study. We identified 30 separate publications reporting on 11 trials.

The searches yielded 736 distinct references. Of these, 16 records were duplicates, therefore 720 titles and abstracts were eligible for screening. After removing irrelevant and clearly ineligible studies, we assessed the full texts of 93 studies for eligibility. Finally, 61 studies were excluded, and 30 publications that reported results from 11 unique trials were included in this systematic review and meta-analysis (Fig. 1).

### Study characteristics

Of the 30 publications reporting on 11 trials, four were phase III RCTs [9–11, 13], and seven were phase II single-arm studies. The comparisons in the RCTs included the following interventions: BR versus R-CHOP (1 trial), BR versus R-CHOP or R-CVP (1 trial); I-R versus rituximab (I trial); and B-DRC versus DRC (1 trial). All 11 studies used the World Health Organisation (WHO) criteria for diagnosis of WM [1] and IWWM criteria for response definitions (Supplementary Table S3) [16]. The sample sizes of the studies ranged from 23 to 261, with a male predominance and a median age of patients ranging from 58 to 70 years. Details of the included studies are summarised in Table 2. All relevant outcome measures were reported in most studies except for quality-of-life measures, which were not reported in most trials.

**Table 2:** Study characteristics.

Reference	Study details					Patient characteristics								
	Trial number	Type of study	Treatment	Follow up	Sample size	Median age	Male/female (%)	Signs and symptoms (%)	Median Hb g/dL	Median Plt x10^9^/L	Median IgM g/dL	Albumin < 3.5 g/dL	Median β2-microglobulin mg/dL	IPSSWM risk score
Gavriatopoulou et al., 2017 [19]	NCT00981708	Phase II Single Group Assignment	Bortezomib Dexamethasone Rituximab (5 cycles) Cycle 1 [21d] Single agent Bortezomib (IV 1.3 mg/m2 on D1,4,8,11) Cycles 2-5 [35d] Bortezomib (IV 1.6 mg/m2 on D1,8,15,22) Dexamethasone (IV 40 mg) Rituximab (IV 375 mg/m2)	6 years	59	70	64/36	B symptoms 19 Hyperviscosity 20 Lymphadenopathy 43 Splenomegaly 29	Not reported 82% Hb <11.5 g/dL	Not reported 17% Plt <100 × 10^9^/L	3.86	48%	Not reported 64% β2-microglobulin >3 mg/dL	Low 15% Intermediate 40% High 45%
Ghobrial et al., 2010 [4]	NCT00422799	Phase II Single Group Assignment	Bortezomib Rituximab (Six 28-day cycles IV bortezomib 1.6 mg/m2 on d1,8,15 every cycle. IV rituximab 375 mg/m2 on d1,8,15,22 on cycles 1 and 4)	2 years	26	62.5	58/42	Hyperviscosity 23 Lymphadenopathy 50 Splenomegaly	11.1	242	4.28	Not reported	3.5	Low 38% Intermediate 42% High 12% Unknown 8%
Treon et al., 2009 [6]	NCT00250926	Phase II Single Group Assignment	Bortezomib Dexamethasone Rituximab (Eight 21d cycles Induction: 4 continuous cycles, then 4 additional cycles spaced 12 weeks apart IV bortezomib 1.3 mg/m2 on d1,4,8,11 IV dexamethasone 40 mg on d1,4,8,11 IV rituximab 375 mg/m2 on d11)	22.8 months	23	66	Not reported	Hyperviscosity 22 Lymphadenopathy 17 Splenomegaly	Median Hct 29.8%	198	4.83	Not reported	Not reported	Not reported
Rummel et al., 2013 [10]	NCT00991211 (STiL)	Phase III Non-inferiority	Up to six 28d cycles Bendamustine Rituximab (IV bendamustine 90 mg/m2 on d1,2) Rituximab (IV 375 mg/m2 on d1) Up to six 21d cycles R-CHOP (Rituximab IV 375 mg/m2 on d1, IV vincristine 1,4 mg/m2 d1, up to 2 mg maximum dose, IV doxorubicin 50 mg/m2 d1, IV cyclophosphamide 750 mg/m2 d1, PO prednisolone 100 mg d1-5)	5 years	44	64	Not reported	B symptoms 38 (BR) 29 (RCHOP)	Not reported	Not reported	Not reported	Not reported	2.6 (BR) 2.4 (RCHOP)	Not reported
Flinn et al., 2019 [11]	NCT00877006 (BRIGHT)	Phase III Non-inferiority	6 to 8 28d cycles Bendamustine (IV 90 mg/m2 on d1,2) Rituximab (IV 375 mg/m2 on d1) 6 to 8 21d cycles R-CHOP/R-CVP (RCHOP: Rituximab IV 375 mg/m2 on d1, IV vincristine 1,4 mg/m2 d1, up to 2 mg maximum dose, IV doxorubicin 50 mg/m2 d1, IV cyclophosphamide 750 mg/m2 d1, PO prednisolone 100 mg d1-5) (RCVP: Rituximab IV 375 mg/m2 on d1, IV vincristine 1,4 mg/m2 d1, up to 2 mg maximum dose, IV cyclophosphamide 750 mg/m2 d1, PO prednisolone 100 mg d1-5)		447 -11 (WM)	60 (BR) 58 (RCHOP/R-CVP)	61/39 (BR) 59/41 (RCHOP/R-CVP)	B symptoms 36 (BR) 39 (RCHOP/R-CVP)	Not reported	Not reported	Not reported	Not reported	Not reported	Not reported
Flinn et al., 2017[36]	NCT01029730	Phase II single arm	Six 28d cycles Bendamustine Rituximab Bortezomib (IV bendamustine 90 mg/m2 d1,2 IV bortezomib 1.6 mg/m2 d1,8,15 IV rituximab 375 mg/m2 d1,8,15 (Cycle 1); d1 (Cycles 2-6)	54 months	54 - 4 (WM)	64	52/48	Not reported	Not reported	Not reported	Not reported	Not reported	Not reported	Not reported
Luminari et al., 2013 [12]	NCT01929265 (INFL09)	Phase II single arm	Six 28d cycles Bendamustine Rituximab (IV bendamustine 90 mg/m2 on d1,2 or d2,3) Rituximab (IV 375 mg/m2 on d1) followed by two additional doses of rituximab. (IV rituximab 375 mg/m2)		72 - 32 (WM)	65	65/35	B symptoms 16	Not reported	Not reported	Not reported	Not reported	Not reported	Not reported
Laribi et al., 2019 [21]	FILO	Phase II single arm	Up to six 28d cycles Bendamustine Rituximab (IV bendamustine 90 mg/m2 on d1,2 IV rituximab 375 mg/m2 on d1)	23 months	69	69	67/33	Not reported	9.7	Not reported	2.38	Not reported	3.49	Low 20% Intermediate 46% High 34%
Dimopoulos et al., 2012 [37]	NCT01788020	Phase II single arm	Six 28d cycles Dexamethasone Rituximab Cyclophosphamide (Cycle 1: PO dexamethasone 20 mg d1 IV rituximab 375 mg/m2 d1 PO cyclophosphamide 100 mg/m2 BD d1-5 Cycles 2-6: PO dexamethasone 20 mg d1 SC rituximab 1400 mg d1 PO cyclophosphamide 100 mg/m2 BD d1-5)	23.4 months	72	69	62.5/37.5	B symptoms 24 Lymphadenopathy 39 Hyperviscosity 25 Splenomegaly 32	Not reported 57% Hb <10 g/dL	Not reported 4% Plt <100 × 10^9^/L	3.6	40%	Not reported 43% β2-microglobulin >4 mg/dL	Not reported
Dimopoulos et al., 2018 [13]	NCT02165397 (INNOVATE)	Phase III	Ibrutinib Rituximab vs rituximab vs ibrutinib (open label) IR (PO ibrutinib 420 mg daily IV rituximab 375 mg/m2 weekly x4 weeks, then weekly x4 weeks after a 3 m interval) R (Placebo daily IV rituximab 375 mg/m2 weekly x4 weeks, then weekly x4 weeks after a 3 m interval) I (open label) PO ibrutinib 420 mg daily)	26.5 months	75 (IR) 75 R	70 (IR) 68 (R)	45/55 (IR) 54/46 (R)	Lymphadenopathy 75 (IR) 77 (R) Splenomegaly 12 (IR) 24 (R)	10.5 (IR) 10 (R)	Not reported 5% Plt <100 × 10^9^/L 9% Plt <100 × 10^9^/L (R)	3.29 (IR) 3.18 (R)	Not reported	3.4 (IR) 3.9 (R)	Low 20% (IR) 23% (R) Intermediate 44% (IR) 37% (R) High 36% (IR) 40% (R)
Buske et al., 2023 [9]	NCT01788020	Phase III	Six 28d cycles B-DRC vs DRC B-DRC (Cycle 1: SC bortezomib 1.6 mg/m2 d1,8,15 PO dexamethasone 20 mg d1 IV rituximab 375 mg/m2 d1 PO cyclophosphamide 100 mg/m2 BD d1-5 Cycles 2-6: SC bortezomib 1.6 mg/m2 d1,8,15 PO dexamethasone 20 mg d1 SC rituximab 1400 mg d1 PO cyclophosphamide 100 mg/m2 BD d1-5) DRC (Cycle 1: PO dexamethasone 20 mg d1 IV rituximab 375 mg/m2 d1 PO cyclophosphamide 100 mg/m2 BD d1-5 Cycles 2-6: PO dexamethasone 20 mg d1 SC rituximab 1400 mg d1 PO cyclophosphamide 100 mg/m2 BD d1-5)	27.5 months	204	68	67/33 DRC 68/34 B-DRC	Lymph nodes: DRC 43%, B-DRC 53%, Spleen >13 cm: DRC 33%, B-DRC 23%	9.8(DRC) 10(B-DRC)	Not reporter. Plt <100 14.4% DRC, 17.8 B-DRC.	31.9 (DRC) 31.7(B-DRC)	Not reported	Median not reported. % with B2M > 3 mg/l 74.4(DRC) 31.7(B-DRC)	Low 12% (DRC) 10% (B-DRC) Intermediate 31% (DRC) 44% (B-DRC) High 36% (DRC) 46% (B-DC) 40% (R)

*IPSS* International Prognostic Scoring System, *WM* Waldenstrom macroglobulinaemia, *Plt* platelet, *IgM* immunoglobulin M, *IR* ibrutinib rituximab, *PO* per oral, *BD* twice a day, *SC* subcutaneous, *Hb* haemoglobin, *DRC* dexamethasone, cyclophosphamide, rituximab.

### Meta-analysis

#### Rituximab-based chemo-immunotherapy regimens

##### Response rates and time to best response

**Fig. 2:**
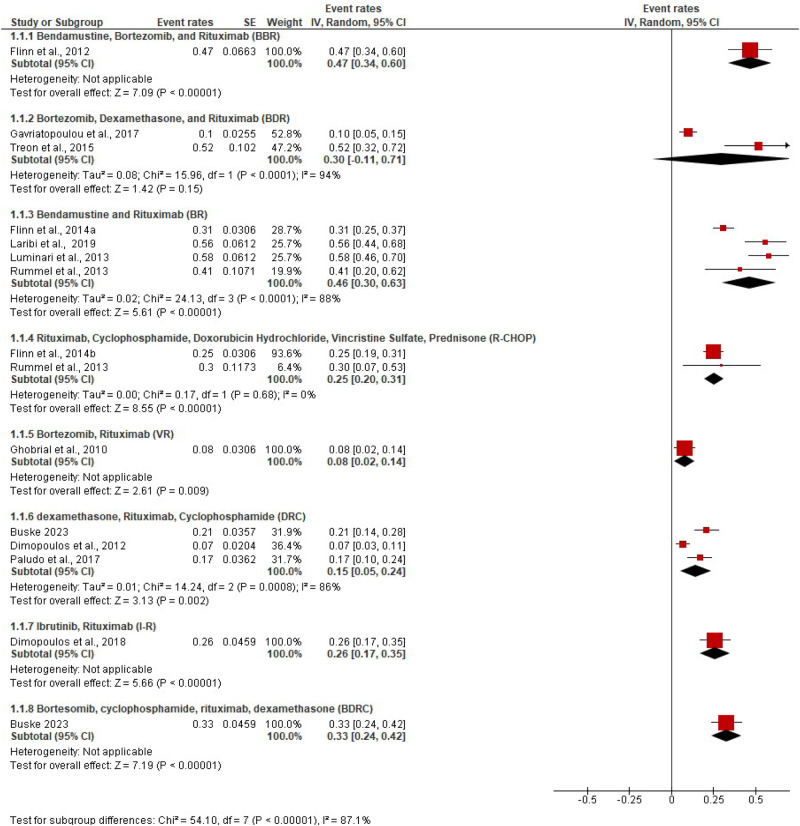
Response rates across trials. Complete, near complete or very good partial response rates for all trials.

Rates of complete response (CR) and very good partial response (VGPR) based on IWWM criteria were reported in all 11 trials that were included. When the response rate was reported by more than one study, proportions were pooled from both RCTs and single-arm trials to find the estimates (Fig. 2 and Supplementary Fig. S1). The response category near CR (nCR) was reported in only one trial [4]. The combined CR, VGPR and nCR rates in patients given rituximab-based chemo-immunotherapy regimens were 47% (95% CI 34–60%) with bortezomib bendamustine rituximab (BBR); 46% (95% CI 30–63%) with BR; 33% (95% CI 24–40%) with BDRC; 30% with BDR; 25% with R-CHOP; 15% with DRC and 8% with bortezomib rituximab.

**Fig. 3:**
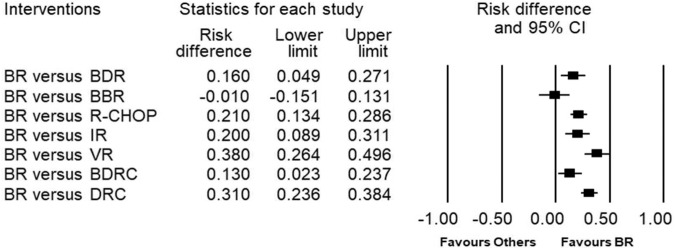
Risk difference for response rates between regimens. The risk difference for complete, near complete response, very good partial response or partial responses.

A comparison of regimens showed that bendamustine-based therapies (BR and BBR) had a higher probability of achieving a CR or VGPR than other regimens (Fig. 3). Two RCTs (Rummel et al., 2013 [10], Flinn et al., 2014 [17]) compared BR versus R-CHOP or R-CHOP/R-CVP respectively and the pooled evidence shows that response rates were higher with BR; however, the risk difference was not statistically significant (RD 0.06, 95% CI −0.02 to 0.14). Similar results were observed with one RCT that compared B-DRC to DRC (Buske et al., 2023 [9]). The evidence suggests that patients in the B-DRC arm had higher rates of CR or VGPR than those in the DRC group, although the difference observed was not statistically significant (RD 0.12, 95% CI −0.01 to 0.24).

Overall pooled major response (combined CR, VGPR, PR) rates for each regimen were as follows: BBR (89%), BR (83%), BDR (82%), Bortezomib, Rituximab (66%), DRC (81%), BDRC (85%) and RCHOP (91%) (Supplementary Fig. S4). PR rates for each regimen (pooled values for regimens evaluated in more than one trial) were as follows: BR (44%), BBR (42%) BDR (54%), Bortezomib, Rituximab (58%), DRC (71%), BDRC (53%) and RCHOP (66%) (Supplementary Figs. S2 and S3).

The median time to best response was 6.8 months for DRC [18] and five [19] to 15 months [6] for BDR. Median time to best response was not reported in the trials evaluating BR. The median time to first response was 1.4 to 3 months for BDR [6, 19] and 3.3 months for B-DRC [9]. As the time to response data were not consistently reported across trials, a meaningful comparison between regimens was not possible in this case.

### Progression-free survival

**Fig. 4:**
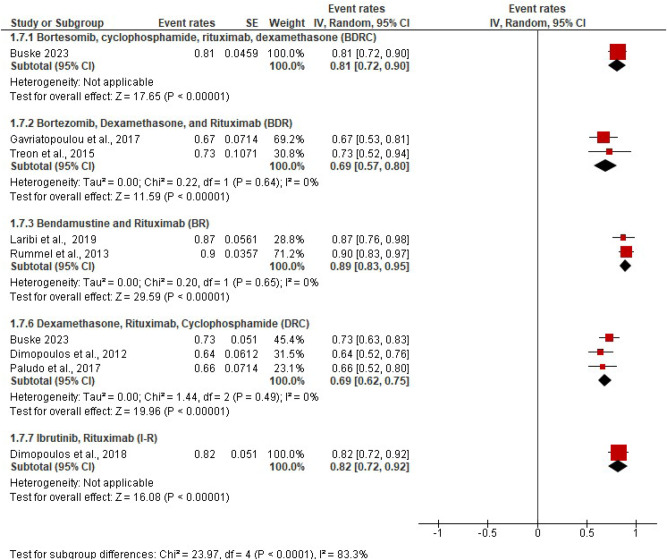
Progression-free survival. Two-year progression-free survival in trials where it was reported.

Two-year pooled PFS rates for each regimen were as follows: BR (89%), BBR (89%), BDR (69%), Bortezomib, Rituximab (66%), DRC (69%), Bortezomib-DRC (81%) (Fig. 4). Evidence from one trial evaluating the BBR regimen reported a PFS of 75% at 3 years [12]. Five-year PFS was reported for three regimens: BDRC (63%), BR (74%) and DRC (32%) (Supplementary Fig. S5). These data suggest that Bendamustine-based regimens result in better PFS at 3^-^ and 5^-^ years compared to other rituximab-based chemo-immunotherapy regimens.

### Overall survival

The duration of follow-up and reporting of OS varied across trials. However, 2-year OS rates were reported by six trials and the available data were combined when two or more trials reported this outcome to evaluate the pooled estimate. The 2-year OS rates reported were as follows: BR (97%), BDR (80%), DRC (91%), BDRC (94%) (Supplementary Fig. S6).

### Comparing BTK inhibitor-based regimens with rituximab-based immuno-chemotherapy

The major response rate for patients treated with IR was 73%, and the median time to best response was 3 months (range 1–46 months) [20]. The combined CR and VGPR rate for IR was 26% (95% CI 17–35%) compared to 47% (95% CI 34–60%) for BBR; 46% (95% CI 30–63%) with BR; and 33% (95% CI 24–40%) with BDRC (Fig. 2). IR resulted in 2-year PFS (Fig. 4) and OS (Supplementary Fig. S6) of 82% and 90%, respectively [20] which is comparable to the rituximab-based immuno-chemotherapy regimens overall.

### Outcomes based on MYD88 and CXCR4 mutational status

Outcomes based on MYD88/CXCR4 mutational status were not reported in most trials. A phase II trial of BR showed no significant difference in response rates or PFS based on MYD88 or CXCR4 mutational status [21]. It is however noteworthy that the numbers of patients in each mutational category were small, and none of the MYD88/CXCR4 double mutant patients progressed, while two of six double wild type patients did. The INNOVATE trial also reported similar response rates and PFS in patients treated with IR across mutational subtypes [13, 20]. It is noteworthy that responses to the BDRC regimen also appeared to be independent of MYD88/CXRC4 mutational status [9].

### Treatment-related adverse effects

In terms of toxicities, IR was associated with more grade 3 and 4 cardiac/vascular toxicities, including hypertension and arrhythmias [13, 20]. Bortezomib-containing regimens were associated with an increased incidence of peripheral neuropathy, with approximately 20% and 10% of patients experiencing at least grade 2 and grade 3-4 neuropathy, respectively [4, 6, 7, 19]. It is noteworthy that more recent studies using subcutaneous bortezomib reported a lower incidence of peripheral neuropathy (10% grade 1 and 2% grade 3) [9]. Chemotherapy-based regimens, such as DRC, were associated with a higher incidence of haematological toxicity than those using bortezomib and ibrutinib. Of note, 20% of patients receiving DRC [8, 18] and 29% of those receiving BR [10, 11] experienced grade 3-4 neutropenia, compared to approximately 12% in those receiving bortezomib-based treatments [4, 6, 7, 19] and 10% of those receiving IR [13, 20].

## Discussion

We present the first systematic review and meta-analysis comparing first-line treatment regimens for WM in the BTKi era. Current practice recommendations by the IWWM [2] suggest the use of either rituximab-based chemo-immunotherapy, rituximab combined with proteasome inhibitors, or BTKi, in symptomatic, treatment-naïve patients with WM. In this systematic review, evidence from comparative and non-comparative studies suggests that among the options recommended by the IWWM, bendamustine-based regimens may result in improved response rates and PFS compared to BTKi- and bortezomib-based regimens. It is noteworthy that OS did not differ significantly between the treatment groups, which may be a function of the effective second-line therapies available.

A comparison of treatment regimens for WM had previously been attempted by Santos Lozano et al. [22]. This study did not however include the key RCTs evaluating BR and was performed before the advent of BTKi. More recent studies by Khurshid et al. [23] and Zheng et al. [24] also did not include BTKi in their comparison of treatment options. Of these, Khurshid et al. [23] focused exclusively on purine analogues in combination with rituximab and did not include bendamustine-containing regimens. Zheng et al. [24] found that rituximab, in combination with either proteasome inhibitors or alkylating agents, yielded the best outcome, with overall response rates of 86% in both groups. A meta-analysis comparing first and second-generation BTKi in newly diagnosed and relapsed WM has also been performed, suggesting they are of similar efficacy but differ in their toxicity profiles [25]. A retrospective multi-centre comparison of treatment-naïve WM patients showed that BR resulted in improved response rates but not prolonged survival compared to ibrutinib [26]. These findings are concordant with our analysis.

Unlike previous studies, we did not include purine analogue-based therapy in our analysis as these regimens are not part of the first-line treatment recommendations by the IWWM [2]. We also did not include second-generation proteasome inhibitors or novel anti-CD20 antibodies such as ofatumumab and obinutuzumab for the same reason.

The identification of MYD88 and CXCR4 mutations has been shown to have prognostic and therapeutic implications, resulting in the latest IWWM recommendations that treatment (especially for BTKi) be adapted based on mutational status [2]. The majority of data supporting the impact of mutational status on therapeutic outcomes are in relapsed WM [27]. Results from ongoing studies are eagerly awaited in the first-line setting.

Although this would have provided valuable information for our analysis, we were unable to compare the efficacy of treatment regimens based on mutational status, as most trials did not report this data. It is interesting that the activity of the B-DRC, BR and IR regimens in treatment-naïve WM appears to be independent of MYD88/CXCR4 mutational status. The number of patients in the genomic analyses was however small, and the studies were not powered to answer this question [9, 13, 21]. Nevertheless, these results bring into question the role of MYD88 and CXCR4 mutations as biomarkers of response to therapy. Future studies stratifying patients by mutational subtype will be crucial to address this important question.

We were not able to compare the PFS in patients achieving a CR or VGPR with those achieving a PR as we do not have access to individual patient data from the trials. This would have been an interesting analysis to determine if the depth of response correlates with PFS. Another major limitation of our study, as it was with previous systematic reviews, is the heterogeneity among the existing trials, not just in terms of trial design but also with regard to the reporting of results. Key outcome measures such as PFS, time to next treatment and time to achievement of response were not consistently reported in the published studies, and those that are reported are limited by varying duration of follow-up. As a result, it was not possible to perform a quantitative meta-analysis of these outcome measures. We pooled the clinical trials based on treatment groups; heterogeneity was identified within most groups, likely due to variations in the trial design. Furthermore, the genomic subtypes (based on MYD88 and CXCR4 mutational status) of patients were not reported in several trials, resulting in heterogeneity among the patient characteristics in the studies that are included in this review.

The advanced age of the typical WM patient, as well as the chronic, incurable nature of the disease, means that quality of life (QOL) and treatment-related toxicities are very important considerations when making treatment decisions. Despite the availability of several objective measures of QOL [28], these were not consistently assessed in most studies and is a key area to improve on in future WM trials.

The rapidly expanding treatment arsenal for WM holds great promise for patients [29]. Novel BTKi such as acalabrutinib, zanubrutinib and tirabrutinib are showing promising results, and it remains to be seen how they compare to ibrutinib, especially in MYD88 L265P wild-type patients [30]. Recent RCTs suggest that the novel BTKi have a favourable toxicity profile (cardiac and gastrointestinal toxicity in particular) compared to ibrutinib, although their efficacy appears similar [31].

With regard to bortezomib-based regimens, the incidence of neuropathy is an important consideration, given that even mild grade 1 neuropathies are chronic and often impair QOL. Second-generation proteasome inhibitors (carfilzomib, ixazomib) [32] are emerging as highly effective, neuropathy-sparing agents and will be an important consideration for the future. It is also noteworthy that lenalidomide [33] and everolimus [34] have been shown in small studies to be effective, while the results of ongoing studies are awaited. BCL2 inhibition using venetolcax is under active investigation for WM [35] and is likely to play an important role in the treatment of WM in the near future. The inclusion of these agents as monotherapy or in combination with anti-CD20 monoclonal antibodies in future trials promises interesting results that could change practice.

In conclusion, evidence from this review suggests that BR yields higher response rates compared to the other first-line treatment options recommended by the IWWM. Given the efficacy and favourable toxicity profile of the novel BTKi, these may also be considerations for front-line therapy in the near future. The future of WM treatment is however likely to involve a genomically stratified treatment approach. Collaborative, international clinical trials comparing BR against other front-line treatment options in patients stratified by MYD88 and CXCR4 mutational status are called for to definitively identify the optimal first-line treatment for WM.

### Supplementary information

checklist for methods

Supplementary Data

## Data Availability

Data may be shared upon reasonable request by contacting the corresponding author, Dr. Sanjay de Mel, at Sanjay_widanalage@nuhs.edu.sg.
