# Supplementary material for: Efficacy and safety of front-line treatment regimens for Waldenstrom macroglobulinaemia: a systematic review and meta-analysis
Source: Blood Cancer J. 2023 Sep 7;13(1):140. doi: 10.1038/s41408-023-00916-5 (PMC10485051; doi:10.1038/s41408-023-00916-5)

**Supplementary Data**

Supplementary Methods

Databases searched to identify relevant studies

We performed literature searches through the following electronic databases:

- MEDLINE and MEDLINE In-Process (using PubMed platform)
- Embase (using Elsevier Platform)
- The Cochrane library, including the following:
  - The Cochrane Central Register of Controlled Trials
  - The Cochrane Database of Systematic Reviews
  - Database of Abstracts of Reviews of Effectiveness

To identify ongoing, discontinued and completed clinical trials the following websites are also searched:

- ClinicalTrials.gov: <http://clinicaltrials.gov>

- International Clinical Trials Registry Platform: <http://www.who.int/ctrp/en/>

Supplementary Tables

| Criteria | Included | Excluded |
| --- | --- | --- |
| Population | - Adult (≥ 18 years) subjects with waldenstrom macroglobulinemia - All subjects must have been diagnosed accordingly to the WHO criteria - All subjects must have been untreated, who are receiving first-line therapy. | - Children with other B cell lymphoid neoplasms - Subjects who have already received treatment - Subjects who have relapsed - Subjects who have received unapproved drug dosages |
| Interventions | - Rituximab-bendamustine - Rituximab-bortezomib - Rituximab-bortezomib-dexamethasone   Interventions of interest in this review consist of all therapy versions of the listed treatments (monotherapy and combinations) at EU/FDA/HSA/TGA approved doses compared to a placebo or any other active treatment | - Drugs not approved by EU/FDA/HSA/TGA for B-cell lymphomas. |
| Study design | - Randomized, controlled, prospective clinical trials - Nonrandomized, controlled, prospective clinical trials - Long-term follow-up studies (e.g., open-label follow-up studies) - Prospective observational studies (e.g., phase 4 studies) - Systematic reviews (including meta-analyses) - Retrospective cohort studies   **Note:** Nonrandomized clinical trials will be identified at level 1 screening but will be excluded at level 2 or prior to data extraction if the researchers find adequate information from randomized clinical trials for the meta-analysis | - Preclinical studies - Phase 1 studies - Prognostic studies - Retrospective studies - Case reports - Commentaries and letters (publication type) - Consensus reports - Nonsystematic reviews |
| Language | - All languages | - None |
| Date | - Jan 2007 to April 2020 | - Studies published prior to Jan2007 |

Supplementary Table 1. Criteria for the Inclusion and Exclusion of Studies During the Initial Screening Process.

| Criteria | Included | Excluded |
| --- | --- | --- |
| Population | - Same as Table 1 | - Same as Table 1 |
| Interventions | - Same as Table 1 | - Same as Table 1 |
| Comparators | - Same as Table 1 | - Same as Table 1 |
| Outcomes | Primary outcomes   - Progression free survival - Overall survival - Response rates   Secondary outcomes   - Rates of relapses - Rates of treatment failures - Rates of complications - Quality of life scores - Time to achievement of partial remission. - Time to achievement of complete remission. |  |
| Study design | - Randomized, controlled, prospective clinical trials   **Note:** If the researchers find adequate information from randomized clinical trials, data will not be extracted from long-term follow-up studies and prospective observational studies | - Nonrandomized, controlled, prospective clinical trials - Long-term follow-up studies (e.g., open-label follow-up studies) - Prospective observational studies (e.g., phase 4 studies) - Systematic reviews (including meta-analyses)^a^ |
| Language | - All languages | - None |
| Date | - Jan2007 to March 2023 | - Studies published prior to Jan2007 |

^a^ Systematic reviews and meta-analyses will be used for identification of primary studies and will be included at the level 1 and level 2 screens. A decision on whether to include nonrandomized studies, systematic reviews, and/or meta-analyses in their own right will be reached after reviewing the available evidence.

Supplementary Table 2. Criteria for the Inclusion and Exclusion of Studies During the Full-Text Review Process

| **Response** | **Response Criteria** |
| --- | --- |
| Complete response (CR) | Serum IgM values in the normal range  Disappearance of monoclonal protein by immunofixation  No histological evidence of bone marrow involvement  Complete resolution of lymphadenopathy/splenomegaly if present at baseline |
| Very good partial response (VGPR) | At least 90% reduction of serum IgM from baseline or serum IgM values in normal range  Reduction in lymphadenopathy/splenomegaly if present at baseline |
| Partial response (PR) | At least 50% reduction of serum IgM from baseline  Reduction in lymphadenopathy/splenomegaly if present at baseline |
| Minor response (MR) | At least 25% but less than 50% reduction of serum IgM from baseline |
| Stable disease (SD) | Not meeting criteria for CR, VGPR, PR, MR, or progressive disease |
| Progressive disease (PD) | At least one of the following:  A ≥25% increase in serum IgM with a total increase of at least 500 mg/dL from nadir  Confirmation of the initial IgM increase is required when IgM is sole criterion for PD   - Appearance of new lymph nodes >1.5 cm in any axis, ≥50% increase from nadir in sum of product of diameters of one or more node, or ≥50% increase in longest diameter of a previously identified node >1 cm in short axis - Appearance of new splenomegaly or ≥50% increase from nadir in enlargement of the spleen - Appearance of new extra nodal disease - New or recurrent involvement in bone marrow - New symptomatic disease (based on presence of malignant pleural effusion, Bing Neel syndrome, amyloidosis or light chain deposition disease, or other paraprotein-mediated disorder |

**Supplementary Table 3.** Outcome definitions based on International Waldenstrom Macroglobulinaemia workshop criteria (16).

**Supplementary Figures**

Figure S1: Complete, near complete or very good partial response rates for randomised controlled trials.


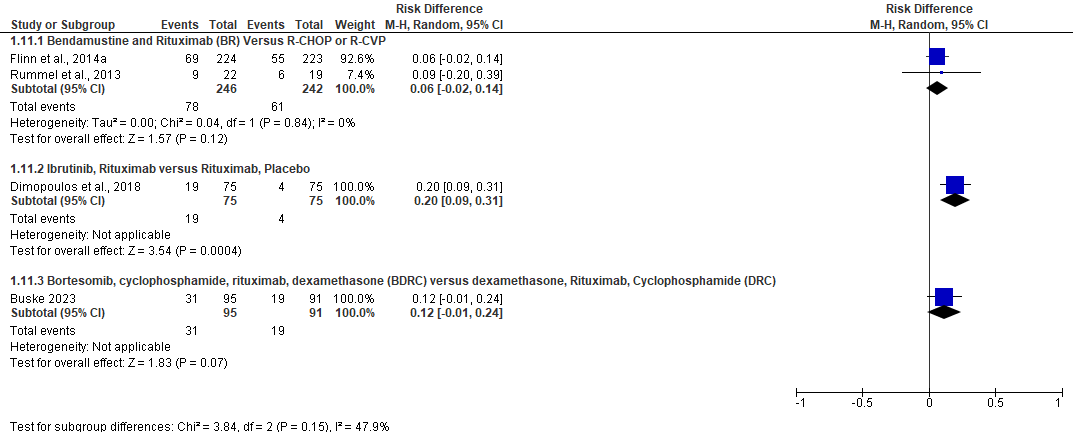


Figure S2: Partial response rates across all trials.


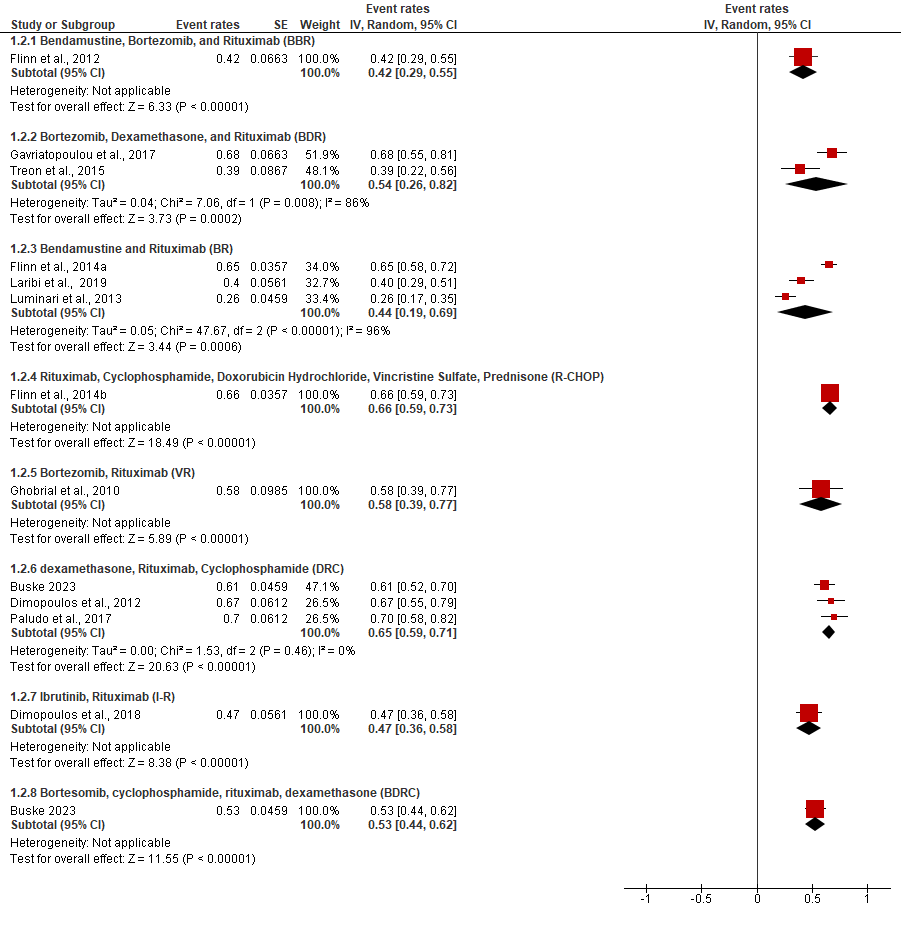


Figure S3: Partial response rates in randomised controlled trials.


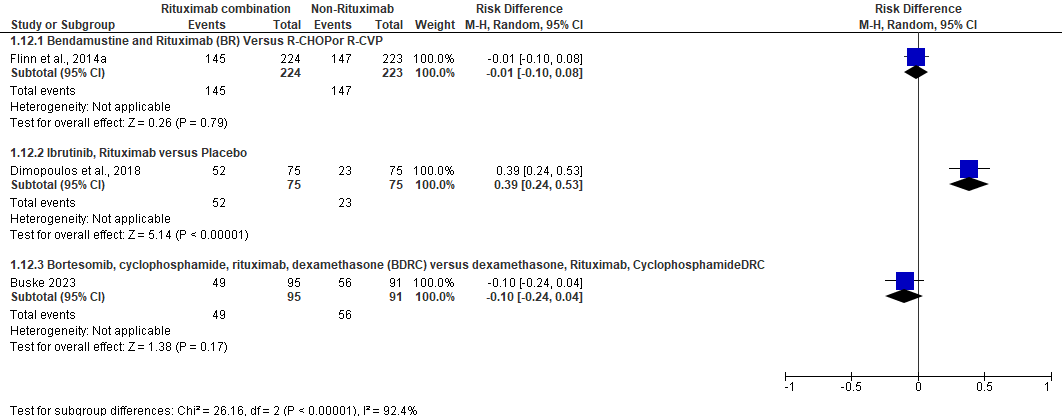


Figure S4: Major Response Rates for all trials


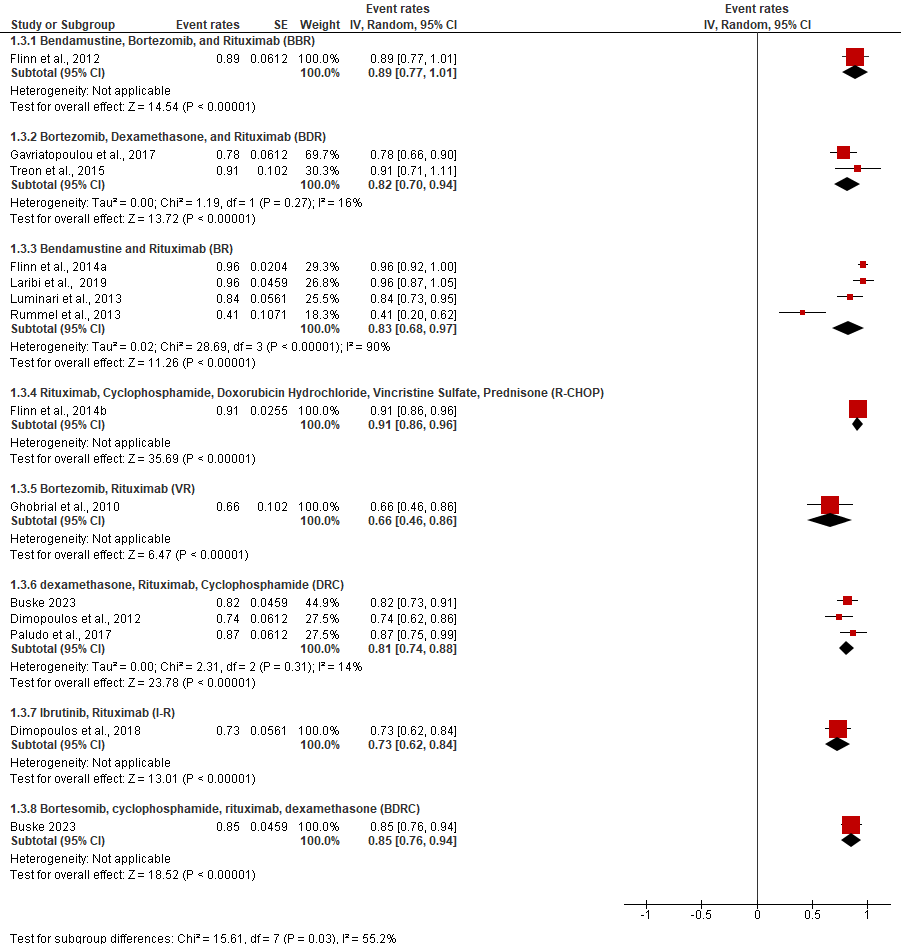


Figure S5: Five-year Progression Free Survival in the trials where it was reported.


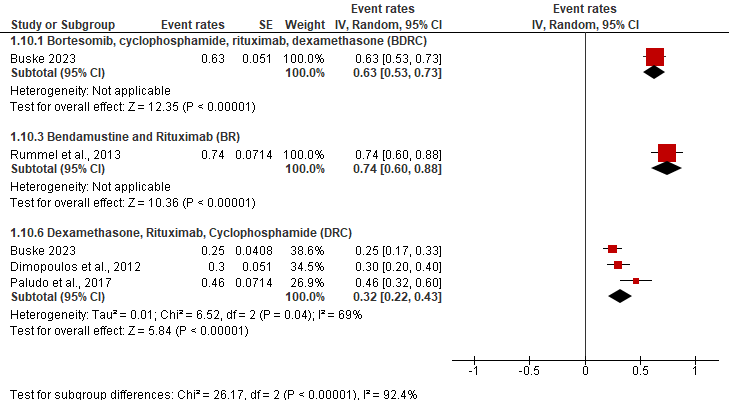


Figure S6: Two-year Overall Survival in the trials where it was reported.


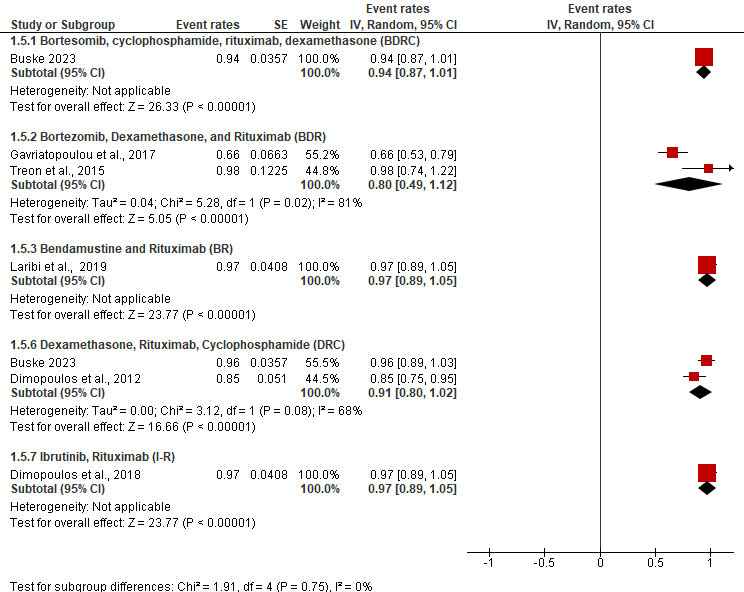

Supplement: Supplementary file 2 — Supplementary Data [file 41408_2023_916_MOESM2_ESM.docx]
